# Authors’ Response to Peer Reviews of “Predicting Waist Circumference From a Single Computed Tomography Image Using a Mobile App (Measure It): Development and Evaluation Study”

**DOI:** 10.2196/53817

**Published:** 2023-12-12

**Authors:** Abderrahmen Masmoudi, Amine Zouari, Ahmed Bouzid, Kais Fourati, Soulaimen Baklouti, Mohamed Ben Amar, Salah Boujelben

**Affiliations:** 1 Surgery Department, Habib Bourguiba University Hospital, Sfax, Tunisia

**Keywords:** waist circumference, computed tomography, abdominal CT, mobile health, health apps, CT, CT scan, CT image, mobile app, app, application, waist, body, body mass, body mass index, morbidity, mortality, clinical, tool, prototype, design, obesity, abdominal usability, validity, medical, BMI

*This is the authors’ response to peer-review reports for “Predicting Waist Circumference From a Single Computed Tomography Image Using a Mobile App (Measure It): Development and Evaluation Study.”*

## Round 1 Review

I would like to thank you for your important comments and questions. Please accept my finest greetings and my humble responses.

I will be answering each comment separately.

### Reviewer K [1]

1. *What was the primary reason for selecting equation 1 as a reference method for waist circumference (WC) calculation? Isn’t it possible to calculate the exact circumference from the computed tomography (CT) images using image-processing algorithms? Wouldn’t it be more representative compared to the manual WC detection procedure?*

Response: Yes, indeed calculating the circumference using CT scan images has already been validated through many papers; however, what our study [2] is trying to do is create a simple and easy tool to retrospectively evaluate the WC using images from CTs, even real images from existing CT radio film papers (with a scale on it).

This method is very simple and easier for nonradiologists (taking a photo with the app and doing an estimation immediately, not waiting for a radiologist with experience in measuring WC with CT software).

The math formula used is a formula of an ellipse; the abdominal perimeter was estimated using the formula as validated by Ciudin et al [3] in their paper.

2. *Keeping the mobile app aside, how much different is this study compared to Ciudin et al [**3**]?*

Response: The study by Ciudin et al [3] compared a regular measurement of WC using the usual method with CT (drawing the perimeter of the abdomen manually with the CT scan software) and the estimation with the formula of an ellipse. We only used this result to estimate the WC in the mobile app, which is very different from using CT scan software. We also performed WC measurement on 10 healthy candidates using both the conventional tape method and the ellipse formula (with the mobile app). We then used a simple linear regression analysis to adjust the final WC formula to the gender of the patient. So our results are adjusted to gender and are established with the mobile app, not the CT scan software—very different.

3. *On page 3, please expand the discussion on “App Requirements.” It is not evident what was meant by “app requirements” in this section.*

Response: The app requirements are the ellipse formula, required measurements (a and b), final formula applied to gender, and the needed parameters and organization of the steps required by the physician to ameliorate the user experience. The text has been modified to further clarify this point.

4. *How many images were taken from each CT slice? As the measurements for the waist parameters (a and b) were taken using a manual process, what kind of procedure was followed to ensure that the person-to-person variability remains low?*

Response: Thank you for your comment and question.

Using the camera of the phone, the app used the CT scan image on the last slice, from cranial to caudal, not showing the iliac bone. The final goal was always to minimize time and make the method as rapid and simple as possible, so each time the first picture was satisfying and clear enough to be used, it was used. To minimize the variability in measurements (wideness of the screen of the phone, wideness of the finger of the user, and personal variability), we specifically used only two variable parameters (a and b). We also only used the CT scan image on the last slice, from cranial to caudal, not showing the iliac bone.

I want to remind you that, even if the precision of the measurement is very important, classifying the patient (with or without abdominal obesity) is the more important result to get from our app, and the small variability in the measurements does not affect it.

5. *In Figures 3 and 4, there is a small dot around the top of the figures. Is this a data point? Additionally, proper x- and y-axis labels are missing. Please add appropriate units on the x- and y-axis.*

Response: Yes, that is a data point; it does not represent a patient, but a difference of means of the measurements. The x-axis of the plot displays the average measurement of the two methods, and the y-axis displays the difference in measurements between the two methods.

The three lines also shown in the plot represent:

1. The average difference in measurements between the two methods,
2. The upper limit of the 95% CI for the average difference, and
3. The lower limit of the 95% CI for the average difference.

The horizontal line drawn in the middle of the chart shows the average difference in measurements between the two methods. This value is often referred to as the “bias” between the instruments.

The further this value is from zero, the larger the average difference in measurements between the methods.

In our case the Bland-Altman analysis showed a mean difference of 0.03 cm between the two measurements, which is very close to zero, indicating that our method using the mobile app is probably reliable.

The units are in centimeters, but with the Bland-Altman test, statistics specialists do not show the unit because it is a representation of how much the two methods of measurement are in accordance.

6. *In the Discussion section, it was claimed that “this is the first of a kind mobile app helping physicians to estimate WC.” Do the authors think the physicians would be able to use apps such as [**4**]* *to assess WC?*

Response: There are many mobile apps to do measurements; we are not reinventing it, but our app is specifically designed to do measurements and apply a unique formula (applied to gender).

I would like to remind you that our app indicates a WC estimation, but the most important parameter is abdominal obesity, so even if the estimated WC does not match the real WC (conventional tape method) in extreme cases, we have an accuracy of 83% when using the mobile app–based WC measurement (mWC) to detect abdominal obesity.

7. *In the Discussion section, it was stated that “Moreover, the simplicity of the app may reduce the time required for physicians to assess WC.” How fast is the app compared to the manual approach?*

Response: Conventional measuring of WC does not require too much time, but it requires the presence of a patient with the app; for any patient who has had a CT scan, the evaluation becomes feasible and easy (retrospectively).

Assessing WC using the conventional methods takes time and expertise for a radiologist; with the mobile app, even a CT image from the patient folder (even on paper or in old CT films) can make the measurement very easy, feasible, and reliable.

#### Minor Comments

8. *The authors stated that “WC cannot be physically assessed in patients with intellectual or motor disabilities” but did not provide any other details as to why it can’t be assessed. The authors should discuss this in detail in the Introduction.*

Response: Taking a conventional tape WC measurement in patients with intellectual or motor disabilities can be challenging. Conventional measurement with tape requires a standing up position and a cooperating understanding patient.

The sentence was modified in the Introduction.

9. *The sentence “However, for a radiologist, this method requires training and can be more or less time consuming” seems confusing. If possible, please restructure this sentence.*

Response: Modified to “However, for a radiologist, this method may require time and training.”

10. *In equation 1, what is denoted by “p”?*

Response: P (perimeter)=WC; this was modified in the text.

11. *Although the authors discussed in the Methods section how the measurements were taken just above the iliac crest and the CT images were taken from the last slice to ensure that those are not taken from different places, do the authors think that there could be some positional errors being introduced based on your approach?*

Response: Maybe yes, but even with the positional errors, the goal of the measurement is not only to have an estimation of the WC but also more importantly to assess abdominal obesity (more important than the exact WC).

12. *On page 3, it was stated that there were further modifications to the app design. What kind of modifications were carried out? Did the authors discard the prior mWCs after modifying the app?*

Response: No, only the design and organization of the steps required by the physician to ameliorate the user experience were modified.

13. *Please try to make sure that periods and commas are being used appropriately. On page 4, one of the sentences was “The mean BMI was 26±4; 27,8±2,7 for women and 24.2±4,4 for men.” For women, a comma was used as a decimal point. On the other hand, for men, a period was used as a decimal point.*

Response: Thank you for your comment. Corrected.

14. *In Table 1, what is the unit for “Confidence Interval”?*

Response: We do not usually express the units; it refers to the mean difference, which is in centimeters.

15. *What kind of procedure was used to perform the diagnostic test to detect abdominal obesity? Please discuss this in the Methods section.*

Response: Abdominal obesity is a simple parameter. Abdominal obesity was defined by WC measurements of >102 cm (~40 in) and >88 cm (~35 in) for men and women, respectively.

This is written in the Methods section.

### Reviewer L [5]

#### Major Comments

1. *The authors admit that their conclusion is based on a very small sample of patients. In recommending further studies, the authors should offer specific guidelines, especially with respect to establishing the precision of each measurement modality. The material speaks only to the accuracy, but the plots in Figures 4 and 5 display some significant outliers.*

Response: Thank you for your valuable comment. True, our study is based on a very small sample of patients and that is why we did not write this paper as a validation of the method (mobile app method) but as an introduction to it. We will need a much bigger sample size and specific guidelines indeed, which will be detailed in the next paper (the validation of the method paper).

Even with the outliers, we succeeded in creating this useful tool that may be used as an easier method for physicians. Additionally, the goal of the mobile app was not only to have an estimation of the WC but also, more importantly, to assess abdominal obesity (we have good accuracy in doing that), so in retrospective studies, assessing this parameter may be very useful and important; we can do that using old CT scan images.

2. *The manuscript should present quantitative evidence of the degree to which an ellipse is an accurate representation of the body shape at the waist.*

Response: Thank you for your comment. In the study of Ciudin et al [3], the Pearson test was 0.987 with a mean error of 0.4 cm and the Bland-Altman analysis showed a mean difference of 1.4 cm between the standing and ellipse formula CT evaluation measurements.

I will add the details to our text to assure scientific honesty.

3. *The comment that this technique is important to less developed countries is puzzling considering the simplicity and extremely low cost of obtaining tape measure data prior to treatment.*

Response: This meant that in less developed countries, CT scan electronic archives are not often available or may not exist. So patient folders (like in low-income countries) are still physical (on paper) and contain images of CT scans (radio films) or CDs. So this method becomes very valuable since it gives the physician the opportunity to extract such a valuable parameter (abdominal obesity) retrospectively and from old paper folders and CDs.

4. *The authors claim that the WC cannot be assessed in patients with intellectual or motor disabilities. Why? That hardly seems like a satisfactory reason to subject the patient to the radiation dose of a CT scan.*

Response: The idea is to assess WC in patients who already have abdominal CT scans and certainly not to order a new one to only assess WC.

5. *Were the statistics presented controlled for variations in BMI and the effect of BMI on the body shape at the waist?*

Response: No, there might be positional errors with the effect of BMI on the shape of the waist; the goal of the measurement is not only to have an estimation of the WC but more importantly to assess abdominal obesity (more important than the exact WC).

#### Minor Comments

6. *The WC is a characteristic of the patient. It is not a parameter. The text needs careful proofreading.*

Response: Thank you for your comment. I agree; the valuable parameter is abdominal obesity.

10. *In the Discussion, why aren’t tape measurements of WC routinely made if this characteristic is so important in treatment planning as the authors claim?*

Response: I agree that they should be. Abdominal obesity is an important morbidity risk factor in many medical and surgical specialties.

11. *The comment “Also, for a radiologist, conventional CT scan method requires training and can be more or less time consuming” is puzzling in light of the ease of using a tape measure in pretreatment planning.*

Response: I agree, and I modified it.

12. *“Since smartphones are commonly available even in low- and middle-income countries”—CT scanners are not so prevalent. This is a pointless polemic.*

Response: I agree—removed.

14. *The suggestion of using artificial intelligence (AI) in an upgraded app is hardly compelling without a clear explanation of why the ellipse fitting is of questionable validity.*

Response: I agree that when using an AI-upgraded app, the ellipse formula may not be needed. The AI will assess the WC directly using image analysis technology.

### Reviewer R [6]

*This manuscript is well written. This paper presents an original idea to simplify patient care. It can be generalized to other specialties.*

*No specific comments.*

#### Major Comment

*This mobile app could be used for other measurements.*

## Round 2 Review

I would like to thank you for your comments and questions. Please accept my finest greetings and my humble response.

### Reviewer K

1. *The authors stated that the app has an accuracy of 83% when using the mWC to detect abdominal obesity. Is it sufficient compared to the conventional approaches? Just a simple comparison/comment would suffice.*

Response: Our estimation based on the app is quite accurate. The percentage of 83% is interesting. As said before, in most cases, it is sufficient, but the more we are talking about extreme numbers (WCs), the less accuracy we get. This comparison was added to the paper.

2. *Related to comment 11 of the round 1 review, how much impact can positional errors have in abdominal obesity classification? This can be explained or discussed in the Discussion.*

Response: In the same spirit as the last comment, the accuracy of WC measurement may be altered in some cases. This may be due to the measurement error in the conventional method or to particular body shapes and extreme values of WC. This comment was already added to the paper.

3. *The Figure 3 regression shows that one of the app measurements was (WC_App=120) when the true value should have been around ~65 (standing app difference=55). However, in Figure 4, that point seems to be missing (mean of standing + app ~92, so the difference ~55 should be around ~92 in the Bland-Altman plot). Can you please clarify this? If my calculations are wrong, I am extremely sorry about that.*

Response: Thank you for your comment. Figure 3 shows the Q-Q plot figure that shows the mean of differences between the two measurements. The Q-Q plot showed good overlapping with some dispersion of extreme values, but the difference between both never exceeds +20 or –10.

## Abbreviations

AI: artificial intelligence
CT: computed tomography
mWC: mobile app–based waist circumference measurement
WC: waist circumference
